# *EGFR* Amplification Is a Phenomenon of *IDH* Wildtype and *TERT* Mutated High-Grade Glioma: An Integrated Analysis Using Fluorescence In Situ Hybridization and DNA Methylome Profiling

**DOI:** 10.3390/biomedicines10040794

**Published:** 2022-03-29

**Authors:** Dorothee Hölzl, Georg Hutarew, Barbara Zellinger, Beate Alinger-Scharinger, Hans U. Schlicker, Christoph Schwartz, Karl Sotlar, Theo F. J. Kraus

**Affiliations:** 1 Institute of Pathology, University Hospital Salzburg, Paracelsus Medical University, Müllner Hauptstr. 48, A-5020 Salzburg, Austria; d.hoelzl@salk.at (D.H.); g.hutarew@salk.at (G.H.); b.zellinger@salk.at (B.Z.); b.alinger@salk.at (B.A.-S.); h.schlicker@salk.at (H.U.S.); k.sotlar@salk.at (K.S.); 2 Department of Neurosurgery, University Hospital Salzburg, Paracelsus Medical University, Ignaz-Harrer-Str. 79, A-5020 Salzburg, Austria; c.schwartz@salk.at

**Keywords:** glioma, glioblastoma, *EGFR*, FISH, EPIC DNA methylation analysis

## Abstract

Gliomas are the most common intrinsic brain tumors in adults, and in accordance with their clinical behavior and patients’ outcome, they are graded by the World Health Organization (WHO) classification of brain tumors. One very interesting candidate for targeted tumor therapy may be epidermal growth factor receptor (*EGFR*) amplification. Here, we performed an integrated comparative analysis of *EGFR* amplification in 34 glioma samples using standard fluorescence in situ hybridization (FISH) and Illumina EPIC Infinium Methylation Bead Chip and correlated results with molecular glioma hallmarks. We found that the EPIC analysis showed the same power of detecting *EGFR* amplification compared with FISH. *EGFR* amplification was detectable in high-grade gliomas (25%). Moreover, *EGFR* amplification was found to be present solely in *IDH* wildtype gliomas (26%) and *TERT* mutated gliomas (27%), occurring independently of MGMT promoter methylation status and being mutually exclusive with 1p/19q codeletion (LOH). In summary, EPIC Bead Chip analysis is a reliable tool for detecting *EGFR* amplification and is comparable with the standard method FISH. *EGFR* amplification is a phenomenon of *IDH* wildtype *TERT* mutated high-grade gliomas.

## 1. Introduction

Gliomas are the most frequent intrinsic brain tumor of adults. According to the guidelines of the World Health Organization (WHO) classification of central nervous system (CNS) tumors, gliomas are assigned to CNS WHO Grades 1 to 4 depending on the clinical behavior and patients’ outcome [1,2]. Whilst CNS WHO Grade 1 pilocytic astrocytomas show a relatively benign course and 10-year survival of approximately 95% [3], CNS WHO Grade 4 glioblastoma show a devastating outcome with a 5-year survival rate of only 4–5% [1,4].

Since the publication of the 2021 WHO classification of tumors of the CNS, an integrated diagnosis and a layered report combining morphology and genetic findings were suggested as an integrated part of glioma classification [1]. Thus, the analysis of genomic alterations such as the isocitrate dehydrogenase 1 and 2 (*IDH1*, *IDH2*) and the histone H3 family 3A (*H3F3A*), *HIST1H3B,* and *HIST1H3C* genes as well as analysis of 1p and 19q status are now integrated aspects of glioma classification. Furthermore, the analysis of O(6)-methylguanine-DNA methyltransferase (MGMT) promoter methylation, mutation analysis of the telomerase reverse transcriptase (*TERT*) promoter, and CDKN2A/B testing are essential for evaluation of glioma [1]. Despite intensive research, survival of glioma patients often remains limited, and curative therapy is still lacking for the most common tumor entities (i.e., high-grade astrocytomas) [5,6,7,8,9,10].

A molecular target for individualized patient care is epidermal growth factor receptor (*EGFR*) amplification [11]. *EGFR*, also termed ErbB1 and HER1, is a receptor tyrosine kinase and is part of the ErbB receptor family [12], playing an important role in cell proliferation, differentiation, and motility [12,13]. *EGFR* binds the epidermal growth factor (EGF) and other growth factors such as growth factor-α (TGF-α), heparin-binding EGF (HB-EGF), and amphiregulin. Ligand binding activates the receptor couples to downstream signaling pathways controlling cell proliferation, growth, differentiation, migration, and inhibition of apoptosis [14,15]. Pathogenic *EGFR* mutations and truncations result in ligand-independent signaling, subsequently leading to upregulation of various pro-oncogenic processes, including chronic cell cycle proliferation [15]. *EGFR* gene amplification directly correlated with protein overexpression and activated signaling [16]. 

Epigenomic DNA-methylation profiling is an emerging approach in tumor classification [1,17,18]. DNA-methylation acts as control of gene transcription enabling on/off switching of transcription by demethylation/methylation of CpG dinucleotide sequences in the gene promoter [17,18]. The Illumina Infinium EPIC (850 k) Bead Chip is an advanced tool for profiling approximately 850,000 CpGs in parallel throughout the human genome with a highly streamlined protocol [17,18]. This information is subsequently analyzed in bioinformatical pipelines enabling the deduction of copy number variation profiles and similarity calculations, e.g., random forest trees and principal component analysis [17,18,19,20]. 

Here, we analyzed 34 glioma specimens of WHO Grades I to IV with regard to *EGFR* amplification status using fluorescence in situ hybridization (FISH) and Illumina Infinium EPIC Bead Chip Arrays. Furthermore, we performed an integrated analysis of *EGFR* amplification in the context of other established molecular hallmarks.

## 2. Materials and Methods

### 2.1. Tissue Collection

In this study, we analyzed 34 anonymized tissue samples, entailing 2 pilocytic astrocytomas CNS WHO Grade 1, 2 oligodendrogliomas CNS WHO Grade 2, 2 astrocytomas CNS WHO Grade 2, 3 astrocytomas CNS WHO Grade 4, and 23 glioblastomas CNS WHO Grade 4, and 2 diffuse midline gliomas H3 K27 altered WHO Grade 4. 

The gliomas were assigned to CNS WHO Grades 1 to 4, and integrated molecular profiling was performed according to the 2021 WHO classification of CNS tumors [1]. All tumor samples were provided by the University Institute of Pathology of the University Hospital Salzburg. The samples used in this study were formalin-fixed and paraffin embedded (FFPE). Prior to study inclusion, samples were anonymized according to the ethics guidelines. Details about glioma samples are listed in Table 1.

Routine immunohistochemical (IHC) analysis was performed on a Ventana BenchMark Ultra device (Roche) using Ventana ready-to-use antibodies against GFAP (760-4345), Ki67 (790-4286), PHH3 (760-4591), and *EGFR* (3C6) according to the manufacturer’s protocols. *EGFR* protein expression levels were scored according to Avilés-Salas et al. within a scale of 0 to 3 [21]. 

### 2.2. Molecular Genetic Characterization of Gliomas

Molecular genetic analysis of glioma samples was performed as previously described [22]. Representative tumor tissues with at least 90% of viable tumor cells were microscopically identified. DNA extraction was conducted applying the Maxwell system (Promega) according to the manufacturer’s instructions. Mutational analysis of *IDH1* and *IDH2* genes were performed with the AmpliSeq for Illumina Cancer Hotspot Panel v2 (Illumina) or the AmpliSeq for Illumina Focus Panel (Illumina), respectively, on an Illumina MiniSeq next-generation sequencing device following the manufacturer’s protocols. Identification of mutations within the hot spot loci of *TERT* promoter, *H3F3A*, *HIST1H3B,* and *HIST1H3C* genes were analyzed by Sanger sequencing as described previously [22,23,24]. Homozygous losses of cyclin-dependent kinase inhibitor 2A/B (CDKN2A/B) were assessed by EPIC copy number variation (CNV) analysis according to Capper et al. [18] and in concordance with the guidelines of the 2021 WHO classification [1].

ZytoLight 1p/1q and 19q/19p probe sets (ZytoVision) were applied according to the manufacturer’s protocols to evaluate the 1p/19q codeletion status of *IDH* mutant gliomas. Deletions of 1p and 19q were double-checked applying EPIC CNV profiles to avoid false positive results that may be due to partial 1p and 19q losses. According to the guidelines of the 2021 WHO classification, 1p/19q status was assessed for all *IDH* mutated gliomas, as loss of 1p and 19q is only occurring in *IDH* mutant gliomas [1]. 

### 2.3. Fluorescence In Situ Hybridization (FISH) Analysis

To evaluate the *EGFR* amplification status, we applied the ZytoLight SPEC *EGFR*/CEN 7 Dual Color Probe set (ZytoVision). Slides were reviewed using fluorescence microscopy, and FISH signals for individual probes were recorded. To assess *EGFR* amplification, we calculated the ratio of green (*EGFR*) and red (CEN7) signals. In accordance with French et al., we set the cut-off value for amplification to equal or greater than 2 [25]. Tumors with polysomy for chromosome 7 but without focal amplification of the *EGFR* gene were considered to be *EGFR* non-amplified.

### 2.4. Infinium Methylation EPIC Array Analysis

Methylation analysis of glioma samples was performed using the Infinium Methylation EPIC Bead Chip (Illumina) according to manufacturer’s protocol. Raw data (idat-files) were analyzed using the molecularneuropathology.org bioinformatics pipeline of the German Cancer Research Center (DKFZ) and the current brain tumor classifier [17]. Copy-number variation (CNV) analysis is an integrated part of the molecularneuropatholgy.org bioinformatics pipeline. *EGFR* amplifications were assessed using the generated CNV plots and ImageJ. *EGFR* status was interpreted in accordance to Stichel et al. as being considered amplified if the respective probes showed an intensity of more than 0.6 on the log2-scale from the CNV after baseline correction (relative probe intensity) [26].

### 2.5. Computational Data Analysis

Statistical analysis was performed using Prism 9 (GraphPad) software suite and Microsoft Excel applying Student’s t-test. Statistical significance was assumed for *p*-values < 0.05. Regression analysis was performed using Prism 9 and Microsoft Excel. 

## 3. Results

### 3.1. Fluorescence In Situ Hybridization (FISH) and Infinium EPIC Methylation Bead Chip Analysis Are Equally Valid Methods in Detection of EGFR Amplifications

**Figure 1:**
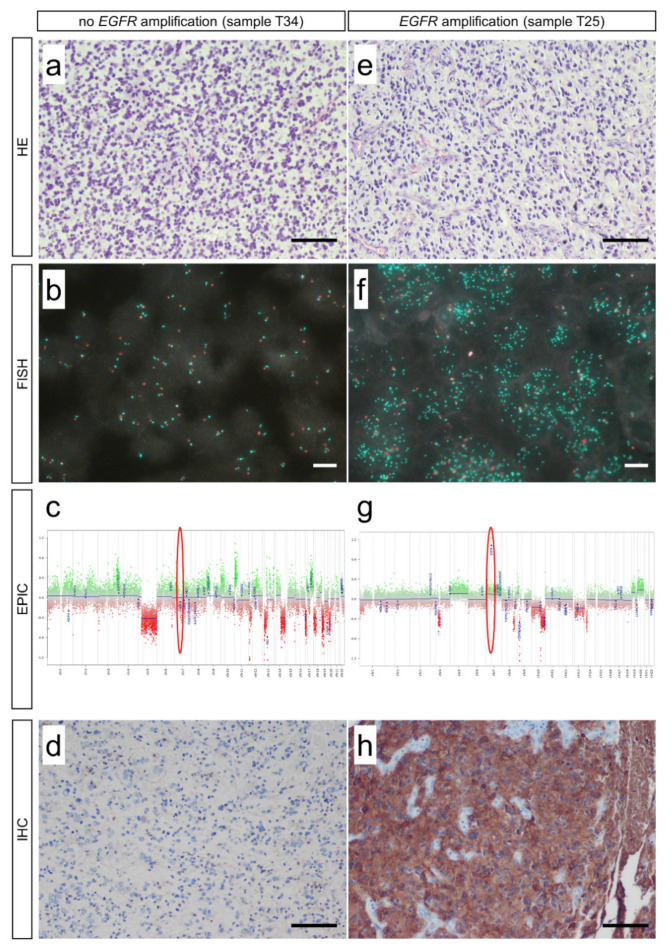
Detection of *EGFR* copy numbers in gliomas. Analysis of 34 glioma samples showed that it is possible to distinguish between *EGFR* non-amplification (e.g., sample T34) (**a**–**d**) and *EGFR* amplification (e.g., sample T25) (**e**–**h**) using FISH (**b**,**f**) and EPIC analysis (**c**,**g**). These findings also correlate with protein expression levels using IHC (**d**,**h**). HE: Hematoxylin–Eosin; FISH: fluorescence in situ hybridization; EPIC: Illumina Infinium EPIC Bead Chip; IHC: immunohistochemistry. Scale bars: 50 µm (**a**,**d**,**e**,**h**), 10 µm (**b**,**f**).

**Figure 2:**
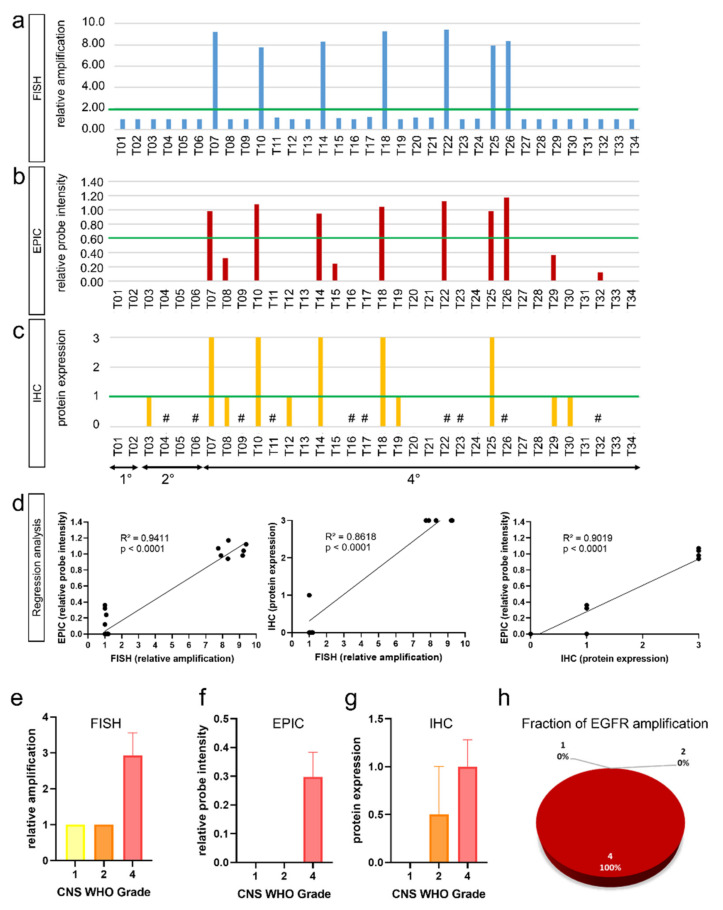
Comparison of two different methodologies for *EGFR* amplification detection. Using both FISH (**a**) and EPIC analysis (**b**), we found seven samples with *EGFR* amplification. Cut-off values for *EGFR* amplification are indicated by a green line and were set according to French et al. to equal or greater than 2 for FISH [25] and according to Stichel et al. to a relative probe intensity of more than 0.6 for EPIC analysis [26]. The results are well in line with protein expression levels detected by immunohistochemistry (**c**) scored according to Avilés-Salas et al. [21]. Regression analysis showed good correlation of *EGFR* amplification using FISH and EPIC analysis as well as FISH and IHC and EPIC and IHC (**d**). Analysis of WHO Grade and *EGFR* status showed that *EGFR* amplification is a hallmark of high-grade CNS WHO Grade 4 gliomas using both FISH (**e**) and EPIC analysis (**f**) as well as IHC (**g**). All gliomas with *EGFR* amplification were of CNS WHO Grade 4 (**h**). #: not performed due to tissue limitation.

In this study, we investigated the validity of *EGFR* detection using conventional FISH analysis and epigenome-wide methylation analysis using the Illumina EPIC Methylation Bead Chip with integrated CNV profiling. DNA methylome analysis by EPIC arrays is a reliable approach in molecular glioma classification [17]. Thus, we performed both FISH and EPIC analysis on all 34 histologically well-characterized gliomas. We found that both FISH and EPIC analysis enabled discrimination of *EGFR* non-amplification and amplification status and that the results are consistent with *EGFR* protein levels assessed by immunohistochemistry (Figure 1a–h). Using FISH, we detected seven gliomas with *EGFR* amplification (Figure 2a). This is in perfect concordance with EPIC analysis that revealed the same seven gliomas being *EGFR* amplified (Figure 2b). Results also correlate with protein expression detected by immunohistochemistry (Figure 2c). Cut-off values were defined as suggested by French et al. (FISH) [25] and Stichel et al. (EPIC) [26]. Protein expression was scored according to Avilés-Salas et al. [21]. Regression analysis showed perfect match of FISH and EPIC analysis (R^2^ = 0.9411, *p* < 0.0001) as well as of FISH and IHC (R^2^ = 0.8618, *p* < 0.0001) and EPIC and IHC (R^2^ = 0.9019, *p* < 0.0001) (Figure 2d). 

### 3.2. EGFR Amplifications Predominantly Occur in High-Grade Glioma

Detailed analysis showed that *EGFR* amplification is unevenly distributed across gliomas. Of all 34 gliomas, we found 7 gliomas (21%) with *EGFR* amplification: While we did not find *EGFR* amplification in low-grade gliomas (0%)—i.e., CNS WHO Grade 1 (0%) and CNS WHO Grade 2 (0%)—25% of high-grade astrocytomas and glioblastomas CNS WHO Grade 4 gliomas showed *EGFR* amplification (Figure 2a–c). Mean *EGFR* gene amplification was 3 in CNS WHO Grade 4 gliomas using FISH (Figure 2e). These results are in concordance with EPIC analysis: We did not find *EGFR* amplification in WHO Grade 1 and 2 gliomas but found mean *EGFR* relative probe intensities of 0.3 in CNS WHO Grade 4 gliomas (Figure 2f). Immunohistochemistry showed mean *EGFR* protein expression of 1 in CNS WHO Grade 4 gliomas (Figure 2g). All identified gliomas with *EGFR* amplification were of CNS WHO Grade 4 (seven cases) (Figure 2h).

### 3.3. Integrated Analysis of EGFR Amplification and Molecular Glioma Hallmarks

Next, we performed an integrated analysis of *EGFR* amplification and molecular glioma hallmarks: *IDH1/2* mutations, *TERT* promoter mutations, MGMT promoter methylation, and LOH 1p/19q.

**Figure 3:**
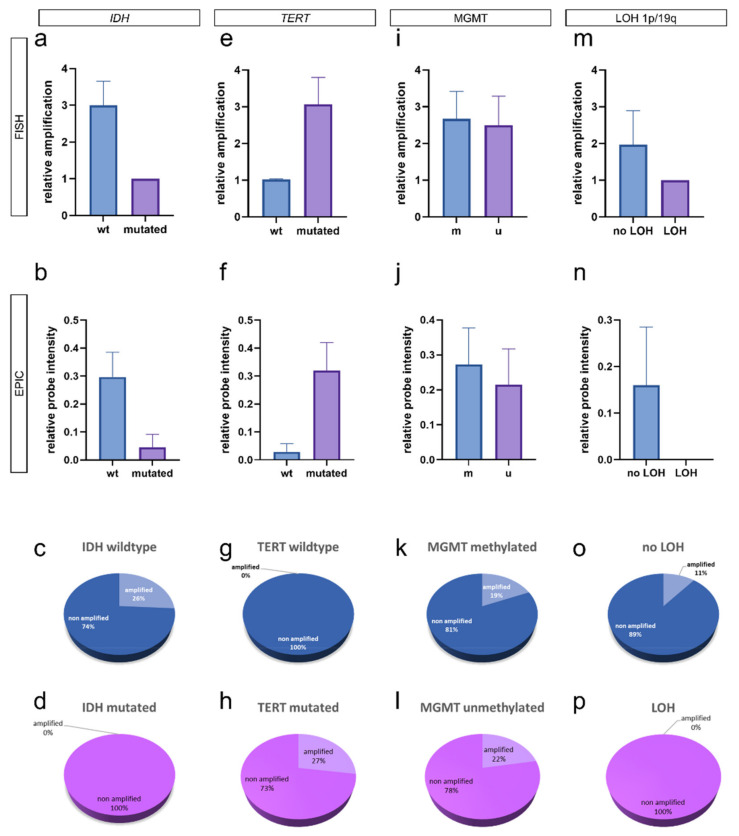
Integrated analysis of *EGFR* amplification and molecular genetic hallmarks of glioma. Correlation of *EGFR* amplification and *IDH* status showed *EGFR* amplification in *IDH* wildtype gliomas using both FISH (**a**) and EPIC analysis (**b**) (*p* > 0.05, Student’s *t*-test), with 26% of *IDH* wildtype gliomas being *EGFR* amplified (**c**) and no *EGFR* amplified case in *IDH* mutated gliomas (**d**). In the case of *TERT* mutation status, we found that *EGFR* amplification occurs in *TERT* mutated cases using FISH (**e**) and EPIC analysis (**f**) (*p* > 0.05, Student’s t-test), with no *EGFR* amplified case in *TERT* wildtype gliomas (**g**) and 27% of *EGFR* amplified cases in *TERT* mutated gliomas (**h**). Analysis of *EGFR* amplification and MGMT methylation status showed no differences in *EGFR* amplification in MGMT methylated and unmethylated gliomas using FISH (**i**) and EPIC analysis (**j**) (*p* > 0.05, Student’s t-test), 19% of MGMT methylated cases (**k**), and 22% of MGMT unmethylated cases showing *EGFR* amplification (**l**). In terms of LOH 1p/19q *EGFR* amplification was found in gliomas without LOH 1p/19q using FISH (**m**) and EPIC analysis (**n**) (*p* > 0.05, Student’s *t*-test), with 11% of cases without LOH 1p/19q (**o**) and no case with LOH 1p/19q being *EGFR* amplified (**p**). wt: wildtype; m: methylated; u: unmethylated; LOH: loss of heterozygosity.

An analysis of *EGFR* amplification and *IDH* mutation status revealed that *IDH* mutated glioma showed fewer copies of *EGFR* using FISH (Figure 3a) and EPIC (Figure 3b) without this being statistically significant (*p* > 0.05, Student’s *t*-test). Of all analyzed *IDH* wildtype gliomas, 26% showed *EGFR* amplification (Figure 3c), and among *IDH* mutated gliomas, none showed an *EGFR* amplification (Figure 3d).

Analysis of *TERT* promoter mutation status and *EGFR* amplification showed that *TERT* mutated glioma show increased copies of *EGFR* using FISH (Figure 3e) and EPIC (Figure 3f) without statistical significance (*p* > 0.05, Student’s *t*-test). Of all analyzed *TERT* wildtype gliomas, none show an *EGFR* amplification (Figure 3g). Of all *TERT* mutated gliomas, 27% showed *EGFR* amplifications (Figure 3h).

An analysis of *EGFR* amplification and MGMT promoter methylation revealed that there is no association between *EGFR* copy numbers and MGMT methylation status using FISH (Figure 3i) and EPIC (Figure 3j) (*p* > 0.05, Student’s *t*-test). Of all analyzed MGMT methylated gliomas, 19% showed *EGFR* amplification (Figure 3k), and 22% of MGMT unmethylated gliomas showed an *EGFR* amplification (Figure 3l).

Analysis of LOH 1p/19q status and *EGFR* amplification showed increased copies of *EGFR* in gliomas without LOH 1p/19q using FISH (Figure 3m) and EPIC (Figure 3n) without being statistically significant (*p* > 0.05, Student’s *t*-test). Of all gliomas without LOH 1p/19q, 11% showed *EGFR* amplification (Figure 3o). Of all gliomas with LOH 1p/19q, none showed an *EGFR* amplification (Figure 3p).

## 4. Discussion

Glioblastomas are the most frequent and most aggressive brain tumors in adults, with a 5-year overall relative survival of only 6.8% [1,27]. One hallmark in glioblastoma therapy was the identification of MGMT promoter methylation that is associated with good therapy response using the alkylating agent temozolomide [8,28,29] and with better outcome [7]. 

A promising target in glioblastoma therapy may be *EGFR* overexpression. *EGFR* inhibition by monoclonal antibodies or small-molecule tyrosine kinase inhibitors (TKIs) has been approved for the treatment of tumor entities such as RAS wildtype colorectal cancers, squamous cell carcinoma of the head and neck (HNSCC), and *EGFR* mutated non-small-cell lung cancer (NSCLC) [11]. 

Here, we assessed *EGFR* gene amplification using FISH and Infinium Methylation EPIC Bead Chip analysis—a technique that is routinely used for molecular brain tumor classifications [17]. We were able to demonstrate, that both FISH and EPIC Bead Chip analysis are equally valid in identifying *EGFR* amplifications: Regression analysis of FISH and EPIC Array revealed very high concordance of both methods for the analysis of *EGFR* amplification (Figure 2d). 

Detailed workup showed that *EGFR* amplification is a phenomenon of high-grade CNS WHO Grade 4 gliomas (Figure 2e–g). Integrated analysis of molecular key hallmarks in glioma (*IDH*, *TERT*, MGMT methylation, and LOH 1p/19q) and *EGFR* amplification showed that *EGFR* amplification is a phenomenon that can be predominantly found in *IDH* wildtype (Figure 3a–d) and *TERT* mutated (Figure 3e–h) gliomas, as well in gliomas without LOH 1p/19q (Figure 3m–p).

Since the importance of *EGFR* amplification has already been established as a precision medicine target in other cancers, such as colorectal cancers, HNSCC, and NSCLC [11], our findings may also open new therapeutic approaches in future brain tumor therapy [30,31]. Thereby, our results are well in line with published data: Bale et al. found that *IDH* wildtype gliomas have a higher prevalence of *EGFR* gene amplification and overexpression than *IDH* mutated gliomas [32]. In terms of *EGFR* amplification and *TERT* promoter mutation, our findings confirm published results: Jaunmuktane et al. found that 82.88% of *IDH* and *TERT* wildtype gliomas were *EGFR* non-amplified, while only 17.12% were *EGFR* amplified. Of *IDH* wildtype and *TERT* mutant gliomas, 58.13% were *EGFR* non-amplified and 41.87% were *EGFR* amplified [33]. In terms of MGMT promoter methylation, Bale et al. found that *EGFR* amplification occurred independently of MGMT promoter methylation status [32]. Our data also support the finding that *EGFR* amplification occurs independently of MGMT promoter methylation. Furthermore, Bale et al. stated that *EGFR* amplification was mutually exclusive of codeletion of chromosomes 1p and 19q (LOH) [32]. Our data also support the finding that *EGFR* amplification is mutually exclusive of codeletions of 1p and 19q.

The detection of combined loss of 1p and 19q in *IDH* mutated astrocytoma is an essential aspect in integrated diagnosis according to the 2021 WHO classification [1]. Thereby, it is important to detect whole arm losses of 1p and 19q [1]. Since FISH probes cover only distinct genomic regions, the use of FISH as the only method is a limitation that may lead to false-positive results [1]. Thus, we additionally double-checked 1p/19q losses detected by FISH in CNV profiles of the EPIC results. 

In summary, our findings demonstrate that Infinium EPIC Bead Chip analysis that is routinely applied in molecular brain tumor classification [17] is a reliable technique for detecting *EGFR* amplifications compared with standard FISH analysis. We found that *EGFR* amplification is a phenomenon that predominantly occurs in high-grade glioma. 

## 5. Conclusions

In conclusion, we demonstrated that EPIC Bead Chip analysis is a reliable tool in detecting *EGFR* amplification that is comparable with the standard FISH method. We found that *EGFR* amplification is a phenomenon of *IDH* wildtype *TERT* mutated high-grade gliomas. 

## Figures and Tables

**Table 1:** Details on glioma samples. Indicated are details on analyzed samples including age, sex, *EGFR* analysis, and molecular genetic hallmarks. n.a.: not available, wt: wildtype, u unmethylated, m: methylated, d: deleted, n: not deleted.

ID	Diagnosis	Grade	Age (y)	Sex	FISH	EPIC	IHC	*IDH1*	*IDH2*	1p/19q	*TERT*	*H3F3A*	MGMT	*CDKN2A/B*
T01	Pilocytic astrocytoma	1	38	f	1.00	0.00	0	wt	wt	n.a.	wt	n.a.	u	n
T02	Pilocytic astrocytoma	1	16	m	1.00	0.00	0	wt	wt	n.a.	wt	n.a.	u	n
T03	Oligod. *IDH* mut. 1p/19q codel	2	27	m	1.00	0.00	1	R132H	wt	1p/19q	C228T	n.a.	m	n
T04	Oligod. *IDH* mut. 1p/19q codel.	2	63	f	1.00	0.00	n.a.	R132H	wt	1p/19q	C250T	n.a.	m	n
T05	Astrocytoma *IDH* mutant	2	47	m	1.00	0.00	0	R132S	wt	wt	wt	n.a.	u	n
T06	Astrocytoma *IDH* mutant	2	22	m	1.00	0.00	n.a.	R132C	wt	wt	wt	n.a.	u	n
T07	Glioblastoma *IDH* wildtype	4	47	m	9.20	0.98	3	wt	wt	n.a.	n.a.	n.a.	u	d
T08	Astrocytoma *IDH* mutant	4	37	f	1.00	0.32	1	R132H	wt	wt	wt	n.a.	m	d
T09	Glioblastoma *IDH* wildtype	4	70	f	1.00	0.00	n.a.	wt	wt	n.a.	C250T	n.a.	m	d
T10	Glioblastoma *IDH* wildtype	4	66	m	7.74	1.07	3	wt	wt	n.a.	C228T	n.a.	m	d
T11	Glioblastoma *IDH* wildtype	4	62	m	1.13	0.00	n.a.	wt	wt	n.a.	C250T	n.a.	m	n
T12	Astrocytoma *IDH* mutant	4	45	f	1.00	0.00	1	R132H	wt	wt	wt	n.a.	m	n
T13	Glioblastoma *IDH* wildtype	4	77	m	1.00	0.00	0	wt	wt	n.a.	C228T	n.a.	u	n
T14	Glioblastoma *IDH* wildtype	4	74	m	8.32	0.94	3	wt	wt	n.a.	C250T	n.a.	m	n
T15	Glioblastoma *IDH* wildtype	4	43	f	1.08	0.24	n.a.	wt	wt	n.a.	C250T	n.a.	u	n
T16	Astrocytoma *IDH* mutant	4	38	m	1.00	0.00	n.a.	R132H	wt	wt	wt	n.a.	m	n
T17	Glioblastoma *IDH* wildtype	4	26	m	1.20	0.00	0	wt	wt	n.a.	wt	n.a.	u	n
T18	Glioblastoma *IDH* wildtype	4	69	m	9.25	1.04	3	wt	wt	n.a.	C228T	n.a.	m	d
T19	Glioblastoma *IDH* wildtype	4	63	f	1.00	0.00	1	wt	wt	n.a.	C250T	n.a.	m	d
T20	Glioblastoma *IDH* wildtype	4	32	m	1.15	0.00	0	wt	wt	1p	C228T	n.a.	u	n
T21	Glioblastoma *IDH* wildtype	4	72	m	1.15	0.00	0	wt	wt	wt	C228T	n.a.	m	d
T22	Glioblastoma *IDH* wildtype	4	79	f	9.4	1.12	n.a.	wt	wt	wt	C228T	n.a.	u	d
T23	Glioblastoma *IDH* wildtype	4	75	f	1.00	0.00	n.a.	wt	wt	1p	C250T	n.a.	u	d
T24	Glioblastoma *IDH* wildtype	4	45	f	1.05	0.00	0	wt	wt	n.a.	C228T	wt	m	n
T25	Glioblastoma *IDH* wildtype	4	77	f	7.90	0.98	3	wt	wt	n.a.	C228T	n.a.	u	d
T26	Glioblastoma *IDH* wildtype	4	49	m	8.35	1.17	n.a.	wt	wt	n.a.	C250T	n.a.	m	d
T27	Glioblastoma *IDH* wildtype	4	25	m	1.00	0.00	0	wt	wt	n.a.	wt	wt	u	n
T28	Glioblastoma *IDH* wildtype	4	51	m	1.00	0.00	0	wt	wt	n.a.	C250T	n.a.	m	d
T29	Glioblastoma *IDH* wildtype	4	63	f	1.00	0.36	1	wt	wt	n.a.	C250T	n.a.	m	d
T30	Glioblastoma *IDH* wildtype	4	65	f	1.00	0.00	1	wt	wt	n.a.	C228T	n.a.	m	d
T31	Glioblastoma *IDH* wildtype	4	76	f	1.05	0.00	0	wt	wt	n.a.	wt	n.a.	m	n
T32	Glioblastoma *IDH* wildtype	4	72	f	1.00	0.12	n.a.	wt	wt	n.a.	C250T	n.a.	u	d
T33	Dif. midline glioma H3 K27 alt.	4	38	m	1.00	0.00	0	wt	wt	n.a.	C228T	K27M	u	n
T34	Dif. midline glioma H3 K27 alt.	4	33	f	1.00	0.00	0	wt	wt	n.a.	wt	K27M	u	n

## Data Availability

Not applicable.
